# Understanding the Factors Influencing Chitosan-Based Nanoparticles-Protein Corona Interaction and Drug Delivery Applications

**DOI:** 10.3390/molecules25204758

**Published:** 2020-10-16

**Authors:** Cristina Moraru, Manuela Mincea, Gheorghita Menghiu, Vasile Ostafe

**Affiliations:** Department of Biology–Chemistry, Advanced Environmental Research Laboratory, West University of Timisoara, 300086 Timișoara, Romania; cristina.g.moraru@gmail.com (C.M.); manuelamincea@yahoo.com (M.M.); gheorghita.menghiu@e-uvt.ro (G.M.)

**Keywords:** chitosan, nanoparticles, protein corona, drug delivery

## Abstract

Chitosan is a polymer that is extensively used to prepare nanoparticles (NPs) with tailored properties for applications in many fields of human activities. Among them, targeted drug delivery, especially when cancer therapy is the main interest, is a major application of chitosan-based NPs. Due to its positive charges, chitosan is used to produce the core of the NPs or to cover NPs made from other types of polymers, both strategies aiming to protect the carried drug until NPs reach the target sites and to facilitate the uptake and drug delivery into these cells. A major challenge in the design of these chitosan-based NPs is the formation of a protein corona (PC) upon contact with biological fluids. The composition of the PC can, to some extent, be modulated depending on the size, shape, electrical charge and hydrophobic/hydrophilic characteristics of the NPs. According to the composition of the biological fluids that have to be crossed during the journey of the drug-loaded NPs towards the target cells, the surface of these particles can be changed by covering their core with various types of polymers or with functionalized polymers carrying some special molecules, that will preferentially adsorb some proteins in their PC. The PC’s composition may change by continuous processes of adsorption and desorption, depending on the affinity of these proteins for the chemical structure of the surface of NPs. Beside these, in designing the targeted drug delivery NPs one can take into account their toxicity, initiation of an immune response, participation (enhancement or inhibition) in certain metabolic pathways or chemical processes like reactive oxygen species, type of endocytosis of target cells, and many others. There are cases in which these processes seem to require antagonistic properties of nanoparticles. Products that show good behavior in cell cultures may lead to poor in vivo results, when the composition of the formed PC is totally different. This paper reviews the physico-chemical properties, cellular uptake and drug delivery applications of chitosan-based nanoparticles, specifying the factors that contribute to the success of the targeted drug delivery. Furthermore, we highlight the role of the protein corona formed around the NP in its intercellular fate.

## 1. Introduction

Chitosan is a naturally occurring biopolysaccharide obtained by N-deacetylation of chitin. Chitosan has been widely studied in the pharmaceutical and biomedical fields due to its excellent biological effects including anti-inflammatory [1], antifungal [2], and antimicrobial activity [3,4]. Further advantages of chitosan in the design of pharmaceutical delivery systems are its biodegradability, biocompatibility, non-toxicity, and mucoadhesive capacity [5]. The electrostatic interactions of cationic chitosan with the negatively charged cell membrane make this material especially suitable for pharmaceutical applications.

Chitosan nanoparticles (NPs) have multiple roles in the pharmaceutical and medical areas, such as wound healing [6], drug, gene and protein delivery systems [7]. They are a highly effective pharmaceutical delivery vehicle due to their ability to protect bioactive macromolecules from enzymatic and chemical degradation in physiological environments and during storage [8]. Chitosan NPs also assist the transportation of charged macromolecules across different cell types [9].

NPs have outstanding features such as prolonged systemic circulation, high preferential retention at tumor sites and the ability to overcome P-glycoprotein-mediated multidrug resistance of cancer cells [10]. There are many methods to prepare chitosan-based NPs, such as emulsion-cross-linking [11], emulsion-solvent evaporation coacervation [12], complex coacervation [13], spray drying [14], ionotropic gelation [15,16,17], membrane emulsification [18], covalent conjugation, [19,20], and dopamine polymerization [19].

## 2. Protein Corona

**Figure 1:**
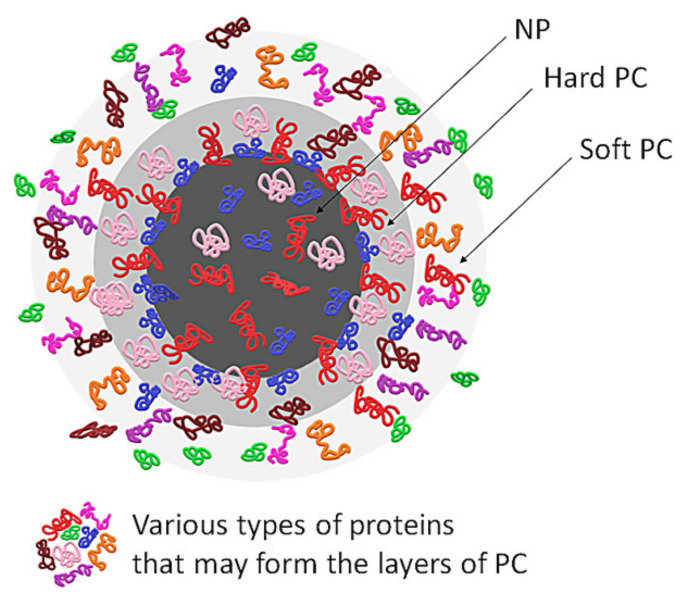
General structure of protein corona. NP—nanoparticle, PC—protein corona.

Nanoparticles entering a biological environment undergo surface modification owing to dynamic physicochemical interactions with proteins and other biomolecules in the extracellular fluids. The adsorption of plasma proteins, lipids and other molecules on the surface of NPs leads to the formation of a stratum of these molecules, commonly called ‘protein corona’ (PC), as the majority of the adsorbed molecules are proteins [21,22] (see Figure 1).

**Figure 2:**
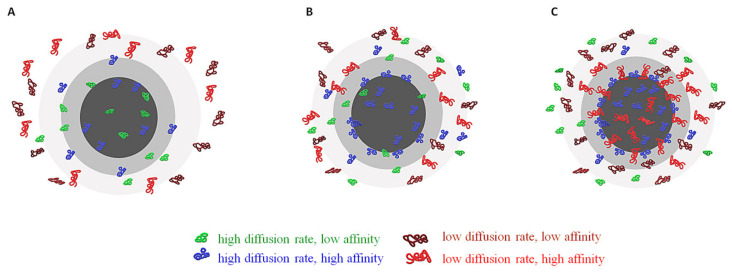
(**A**) Initiation of formation of PC (seconds after the NP reaches the biological fluid); (**B**) Beginning of exchange from the PC of proteins with low affinity with proteins that have higher affinity (seconds to minutes); (**C**) stabilized PC, with proteins with high affinity occupying the first layer of PC (hard PC) and the majority of the second layer (soft PC) where proteins with low affinity are still present.

The chemical structure of the PC and the kinetics of changes of its configuration depend on the affinities of proteins towards the functional groups located on the surface of NPs, but also on the proteins’ concentration. Proteins that adsorb with high affinity form the “hard” corona—which consists of tightly bound proteins that are not easily desorbed—and proteins that adsorb with low affinity form the ‘soft’ corona, which consists of poorly bound proteins [21,22,23] (see Figure 2).

In fact, the proteins that construct the “hard” corona will form the first layer on the surface of NPs in several hours and will have a small desorption rate. The proteins from the “soft” corona interact mostly with the proteins from the “hard” corona, forming the second layer. The “hard” corona does not completely cover the NP’s surface, hence even proteins with low affinity have access to some functional groups located on the NP surface. These “soft” proteins have a higher exchange rate, as their affinities toward the functional groups of NPs surface is low. Even if the proteins’ concentration in biological fluid is very low, the proteins will tend to cover the NP’s surface as much as possible. Virtually, a complete surface coverage of the NP will take place. As “soft” proteins interact with the “hard” corona via weak protein-protein interactions, it may be possible for these interactions to take place even in the absence of NPs in a biological fluid [24]. There are reports of NPs, especially PEGylated ones, that have only a “soft” corona [25,26].

The thickness of the “hard” and “soft” protein corona layers depends on particle size, surface properties, but also on protein concentration and composition. As the hydrodynamic diameter of most proteins present in plasma ranges between 3 and 15 nm, the thickness of the “real” NPs covered with protein corona is too large to be composed of only a single layer. In fact, even the “hard” corona—made by “primary binders” that are supposed to be proteins adsorbed directly on NP surface— and the “soft” corona—made by “secondary binders” that associate with the primary binders via protein-protein interactions—are made from several layers of proteins, depending on the proteins’ concentration in the biological fluid [27]. The exact number of layers and of types of proteins adsorbed on NPs is difficult to count as it depends on the available analytical tool. Roughly 30 years ago the published articles have identified and quantified in a “typical” plasma PC only 2–6 proteins with high abundance and noticed the presence of some other proteins with low abundance. More recently—due to improvement of analytical techniques—the list of proteins identified in protein corona was increased to 125 [24]. On the other hand, the adsorbome—which is the sum of proteins from the PC—is very heterogeneous, especially due to the Vroman effect, i.e., only a small subset of the plasma absorbome is found in the PC from most NPs and only a fraction of the adsorbome proteins binds with high affinity to a specific NP [28]. Among the proteins that seem to be present in the corona of all the studied NPs one may mention albumin, immunoglobulin G, fibrinogen and apolipoproteins [29].

The lifespan of the NP-proteins complex ranges from microseconds to days, depending on the kinetics of association/dissociation which are influenced by the protein concentration and composition of the physiological fluid. In biological fluids with lower protein concentration (like bronchial and ocular fluids) the NP surface will be covered chiefly with lower affinity proteins, while in blood—where the protein concentration is very high—the composition of the protein coating may suffer changes in time due to differences in concentration and affinities of proteins. Shortly after the introduction of a NP in blood, the surface will be covered mostly by serum albumin and fibrinogen, which are present in higher concentration in blood. These proteins may be switched in time with proteins present in a lower concentration but having a higher affinity toward the NP material [22].

The formation of PC on NPs increases their hydrodynamic diameter. If the core of a NP is made from one type of polymer and the shell (cover surface) from another, the increase of the hydrodynamic diameter is more noticeable. For instance, the hydrodynamic diameter of NPs made from PLGA alone increases with 50% due to PC formation and with more than 150 % when the PLGA particle is covered with chitosan before being put in contact with plasma proteins [30]. The PC changes also the zeta potential and so the stability of particles in the plasma environment. If the surface of NPs allows the adsorption of serum albumin, a slight accumulation of negative charges may happen at pH 7.4. If the surface of NPs is covered with chitosan, the increased number of positive charges may attract the proteins that are electronegatively charged, which can conduct an increase of aggregation effect in biological media. If the shell surface of NPs is amphiphilic, the proteins from the formed PC interfere less with the stability of particles as it is mediated by steric repulsion (as in the case of NPs containing pluronics) [30].

Understanding of the PC formation mechanism is essential in predicting the behavior of NPs in biological environments, for nanotoxicology applications and development of drug delivery nanosystems [31,32]. The PC controls the interactions of the NPs with the cells. Endocytosis of NPs may implicate mechanisms of active—receptor-mediated, ATP driven—or passive transport through the cell membrane. The protein corona affects the cellular internalization, the speed of elimination, signaling properties and what tissues the NP is able to penetrate [23,33].

## 3. Physico-Chemical Aspects of the Protein Corona Formation

The critical physico-chemical parameters that influence the formation of the protein corona are the type, size, shape, surface curvature, surface charge, hydrophobicity, polydispersity index and zeta potential. An overview of these parameters and the type of the target cells is presented in Table 1.

### 3.1. Size

The nanoparticle size is an important parameter for all NPs designed for medical applications. Tahara et al. [34] presented an example of the influence of NP size on the cellular uptake using A549 cells. It was clearly demonstrated that the size of NPs must be in submicron range in order to be taken up effectively. In this study conducted on A549 cell lines, the 200 nm NPs proved to have a 2.5-fold greater uptake than 1 µm NPs [34].

The size of chitosan-based NPs prepared by various methods presented in Table 1 is very wide-ranging, from 32.7 [35] to 1100 ± 20 nm [34] depending on the preparation methods and on the targeted application. Some methods produce NPs with a very broad size range, which leads to difficulties in size control.

The use of negatively charged polymers to cover/interact with chitosan-based NPs can alter both the size of the particles and their general charge (zeta potential). For example, when chitosan-based NPs were coated with hyaluronic acid (HA) the size of particles increased from 170 nm to 270 nm and the positive charges of the chitosan polymer were shielded by the negatively charged HA. This effect is even more evident when chitosan-based NPs adsorb alginate (Alg) due to higher charge density of Alg in comparison with HA. At the same time, the size of chitosan-based NPs coated with Alg increased three times in comparison with particles coated with HA [36].

**Figure 3:**
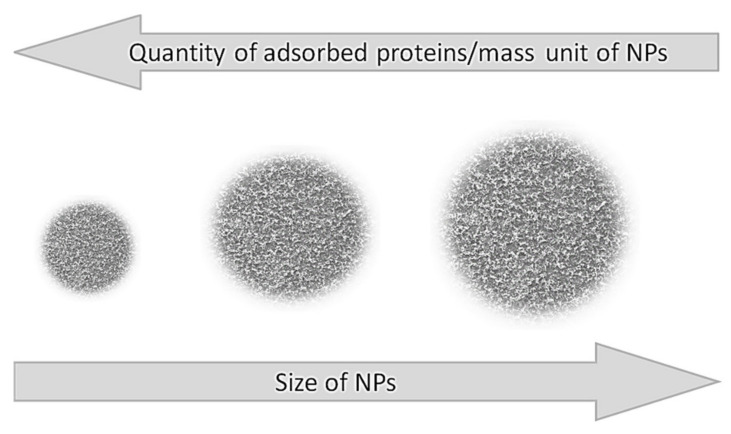
Influence of NP size on the quantity of the adsorbed proteins per mass unit of NP.

Dramatic changes are observed when the size of the NP is approaching the size of proteins. In this case the formation of a second layer of proteins is almost overlooked [37]. As the size of NPs surpasses the size of the enzymes several times, the ratio between the particle size and the amount of protein in the PC is reversed, i.e., the smaller the NP’s size the higher the quantity of protein adsorbed (Figure 3).

For NPs larger than 30 nm there is a direct correlation between their specific surface area and the amount of adsorbed proteins. It was shown that at a constant particle weight, the amount of adsorbed plasma proteins increases with the surface area when the same type of NPs—varying in size between 70 and 700 nm—were used [37]. On the other hand, the thickness of the PC is greater in larger NPs, although the total amount of adsorbed proteins is smaller when compared to the same type of NPs but with a smaller diameter [22] and even small variations in the size of the NP (e.g., 10 nm) can drastically affect the composition of the PC [38].

### 3.2. Surface Curvature

The surface curvature is directly affected by the NP size, which affects the structure of the adsorbed proteins or peptides [49]. NPs of the same or similar surface charge but of different size adsorb proteins to various degrees [50].

**Figure 4:**
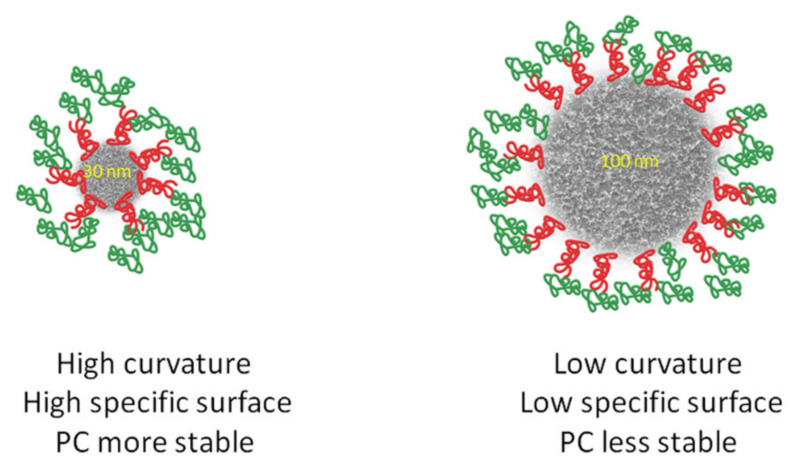
Influence of surface curvature and shape on the stability of the protein corona.

The surface curvature facilitates the adsorption and conformation change of proteins and therefore the composition of the PC [51]. The binding affinities of protein molecules are different for particles of similar compositions but with different curvatures, because the surface curvature of smaller nanoparticles is higher than that of lager particles with the same chemical structure. Protein–protein interactions are also reduced at highly curved surfaces leading to a more diverse composition of PCs. Compared to proteins binding onto the bulkier counterparts of NPs, the composition of the PC bound to a NP with a high surface curvature is more stable [52]. Figure 4 presents the influence of surface curvature and shape on the stability of the PC.

### 3.3. Shape

Nanoparticle shape is another important parameter which controls composition and overall PC formation [52]. The shape of the nanoparticle depends on the method of synthesis. Particles with non-spherical geometry can be fabricated using de novo methods like film stretching or template assembly [53]. It has been demonstrated that non-spherical NPs tumble with the flow while the symmetric spherical NPs move more easily [54]. There are contradictory data on cellular uptake of NPs depending on size. Some studies claim that nanospheres adhere less efficiently to cells as compared to nanorods [55,56], while others show the opposite [57]. This could be due to differences in cell lines or NP coating. Chitosan NPs present globular [39] and spherical [30,42,46,47] shapes in most of the studies. Due to surface curvature, the composition of the PC is different for NPs and flat surfaces immersed in the same biological fluid. Results from flat surface nanomaterials cannot be extrapolated to NPs, and vice versa [21].

### 3.4. Surface Charge

The properties of NPs depend on surface charge. Methods to modify this parameter are used to tailor the final properties of NPs. These modifications may produce also alterations in stiffness that can raise supplementary problems in reproducibility and predictability of properties of final product.

The interactions between the protein molecules and the NP charges have a pronounced influence on the adsorption kinetics and the composition of the PC. The kinetics of the adsorption/desorption process depend on various properties of the NPs and on the chemical composition of the blood or other biological fluids. Roughly, in the initial stage, the molecules having the highest concentration in the biological fluid will be adsorbed on the surface of NP, but later they may be replaced by those molecules (proteins) with higher affinity towards NP surface functional groups [28].

**Figure 5:**
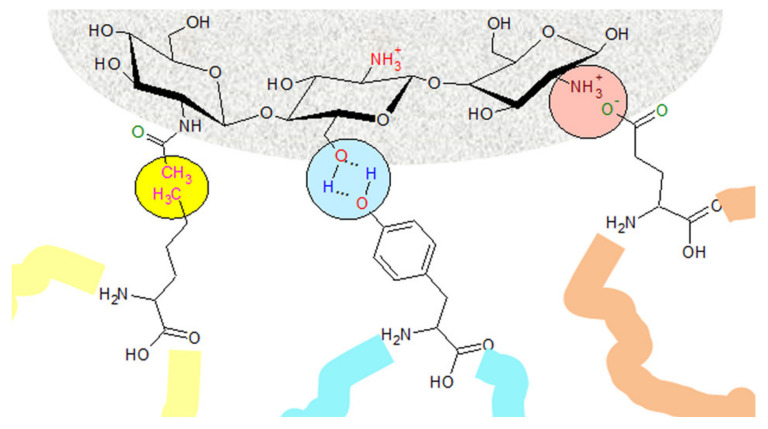
Chemical structure of chitosan on the surface of NPs, and possible interactions with proteins’ amino acids.

It has been shown that negatively charged NPs with a high surface charge density can enhance adsorption of plasma proteins [58]. Particles having basic functional groups with a positive charge were more likely to adsorb proteins with an isoelectric point of less than 5.5 (e.g., albumin), while particles having acidic functional groups—bearing a negative charge—preferentially adsorbed proteins with an isoelectric point higher than 5.5 (e.g., IgG) [29]. Figure 5 shows a possible interaction of various amino acids with the functional groups of chitosan from the surface of a chitosan NP.

After the contact of NPs with plasma, a decrease of charge density with immediate binding of proteins with opposite charges occurs [59]. The corona of chitosan-modified poly(lactic-co-glycolic acid) (PLGA) NPs encountered a maximum electrostatic aggregation effect in the environmental fluid, therefore tended to settle down more [30].

### 3.5. Polydispersity Index

**Figure 6:**
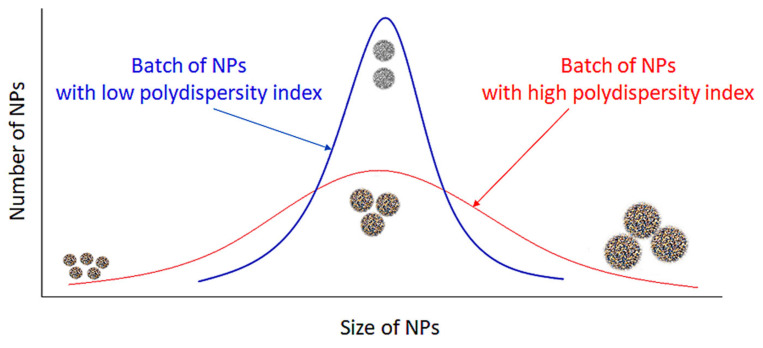
Influence of PDI on homogeneity of PC. A low PDI indicates that the NP solution is homogenous, while a high PDI indicates that a solution is more heterogenous.

The heterogeneity of a sample based on size is measured by the polydispersity index (PDI) (Figure 6). Polydispersity occurs due to size distribution or aggregation of the sample during storage or analysis [60]. The PDI of a polymer is measured by dividing the weight average by number average molecular weight and it ranges from 0 to 1 [61]. The closer to 0 a PDI value, the more homogenous the nanoparticle solution, while values above 0.5 indicate that a NP solution is heterogeneous [62]. The PDI of the chitosan-based NPs presented in Table 1 ranges between 0.009 [39] and 0.48 [46]. As the value of this parameter influences the drug release kinetics and cellular uptake in drug-delivery applications of NPs, it seems important to choose an appropriate preparation method of chitosan-based NPs in order to obtain a low polydispersity and a homogeneous product [63].

### 3.6. Hydrophobic and Hydrophilic Interactions

Hydrophobic and hydrophilic interactions significantly affect and attenuate the adsorption and desorption of proteins over a NP surface. As hydrophobic interactions are always attractive, hydrophobic NPs adsorb more proteins than hydrophilic NPs and are often involved in the denaturation and conformational change of surface-adsorbed proteins [64]. Apolipoproteins have a higher binding affinity for hydrophobic NPs, while fibrinogen, albumin and IgG are more frequently adsorbed by hydrophilic NPs [25]. Hydrophobic particles tend to be opsonized more easily in the blood. This can be prevented by the grafting of hydrophilic polymers onto the NP surface, increasing the duration of its circulation in the bloodstream from a few minutes to several hours [65].

### 3.7. pH

Another essential factor in the interaction of NPs with proteins is the pH. The protein binding affinity of the NP is affected by the change of the environmental pH, and it can lead to a different pattern of protein adsorption. In the cellular uptake pathway nanomaterials may encounter various biological fluids with different pH values, such as blood (pH 7.4), exposure media (pH 6.9–7.4), intracellular fluids (pH 6.8), and lysosomes (pH 4.5–5). Moreover, the acidic environment of tumors contains specific types of proteins which could modify the PC, changing the bioavailability and therapeutic effect of NPs [66]. Chitosan has a pKa value of ~6.5, which makes it establish electrostatic interactions with negatively charged cell membranes [19].

### 3.8. Zeta Potential

**Figure 7:**
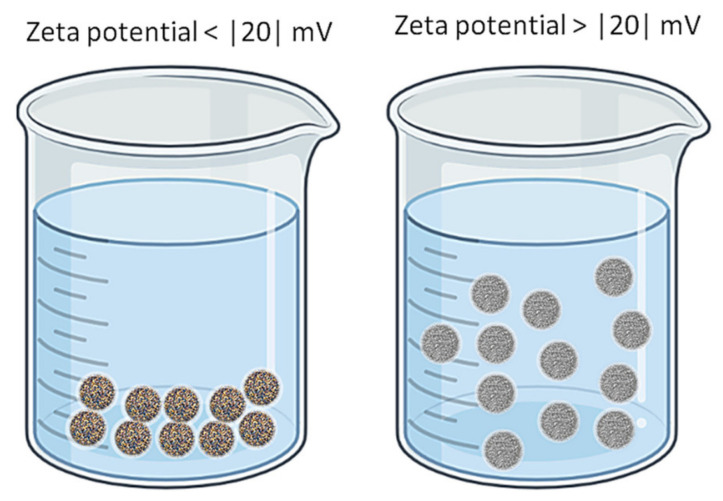
The influence of zeta potential on NP solutions.

One important parameter that characterizes NPs (in fact all colloidal systems) is the zeta potential. Its value can be positive or negative and represents an appreciation of the stability of the NPs in the given environment. At low values of zeta potential the system is unstable and the NPs can cluster and precipitate. As a rule, when the zeta potential is larger than 20 (either positive or negative) there is a good chance that the system is stable, i.e., the NPs are in suspension in the liquid microenvironment [67]. Figure 7 illustrates the influence of the zeta potential on nanoparticle solutions.

Considering the interaction of NPs with cells or cell components, the sign of the zeta potential becomes very important as the binding of NPs to cells can be accomplished by electrostatic interactions. For example, Park, Han [68] reported an experiment when histidine replaced some amino groups of glycol-chitosan NPs and promoted the interactions of these modified NPs with negatively charged endosomal membranes, due to the fact that histidine behaves as a cation at acidic pH values (a property which is enhanced once the NP reaches the lysosomes). A fine tuning of the NPs surface charge using functional groups with pKa values between the normal pH of endosomes and that of lysosomes was conducted in order to influence the targeted delivery of the drug carried by these NPs, either to endosomes, lysosomes or cytoplasm [69]. A similar strategy was also applied when the target cells for drug delivery were cancer cells. Due to the presence of proteoglycans and anionic phospholipids in the inner layer of the cancer cell membrane their surface becomes negatively charged and can interact with NPs bearing positive charges on their surface. When PLGA coated with positively charged chitosan carrying paclitaxel reached the mildly acidic environment of cancer cells, an increased uptake and drug delivery was recorded [70]. This strategy is not limited only to cancer cells. NPs of N-trimethyl chitosan chloride having a positive zeta potential of 30.7 mV were used to deliver an anti-neuroexcitation peptide to brain cells [71]. Adsorption mediated transcytosis of these NPs into cells was possible due to interactions between the positive charges from the surface of particles and negative charges located on the surface of plasmalemmas. Another example of interactions between positively charged chitosan-based NPs with negative charges on cell membranes is presented by Huang and Yang [72].

The types of proteins that will be adsorbed on NPs surface depend on the NP charge and this can be changed if a specific target application is desired. Kim et al. presents an example when PLGA/PVA NPs having a negative zeta potential (−30.1 ± 0.6 mV) were coated with chitosan that led to a positive zeta potential (+26 ± 1.2 mV). The positive charge enhanced the uptake of NPs coated with chitosan by H157 cell types [44].

Is has to be taken into account that when the zeta potential of chitosan-based NPs is measured in phosphate buffers, a shift toward neutral values takes place due to the interaction between positive charges of the chitosan polymer with the negatively charged phosphate ions [73]. In these conditions the measured zeta potential values are close to neutral. However, in body fluids (and in other buffer types) the chitosan-based NPs have a more negative value of zeta potential allowing these particles to show strong mucoadhesivity and interaction with negatively charged surfaces [63].

## 4. Composition of the Protein Corona

The PC composition changes over time depending on the chemical structure and other NP properties. Due to the complexity of interactions between NPs and proteins in the biological fluid, there is no universal PC available for all types of NP and no predictions can be made about the PC structure, even if the chemical composition of the biological fluid is known. We can say that the composition of the protein corona is unique for each type of NP and its determination is an experimental trial and error process. The composition depends on the physicochemical properties of NPs (surface functional groups, surface charges, shape, size, etc.), on the nature and chemical composition of the physiological environment (blood, interstitial fluid, cell cytoplasm, etc.) and the time of exposure and interaction [22].

The “hard” corona structure can last for many hours, enough to influence many biological and physiological cellular processes [21], although there is a continuous competition between the more than 3700 proteins from plasma for adsorption on the same NPs surface sites [37]. As previously mentioned, the kinetic rates of adsorption and desorption of each protein (and lipid) from plasma determines the composition of the protein PC at a certain moment. The kinetics behavior depends on protein abundance and affinity towards the adsorption sites from the NP’s surface. In most of the cases, in the first stage, the corona is formed by those proteins with a higher concentration in the plasma. In later phases these are replaced by those proteins that have a higher affinity, even if their abundance is very low. This process may take several hours. In fact, the fast component is formed in seconds, while the slow component builds on a time scale of minutes to hours. Similarly, the desorption has a mean lifetime of about 10 min for the “soft” corona and about 8 h for the “hard” corona. Due to the Vroman effect [28], the identities of the adsorbed proteins can change over time even if the total amount of adsorbed protein remains roughly constant [22]. Early stages of Vroman process involve the rapid adsorption of albumin, IgG and fibrinogen, which are replaced, in the late stage, by coagulation factors and further by apolipoproteins. It is worth mentioning that the Vroman process is not observed in all situations. In the case of protein adsorption on ultrasmall superparamagnetic iron oxide (USPIO) the results have indicated that there is no typical Vroman effect [74].

## 5. The Fate of the NPs-PC

Upon entering the body, the NPs are recognized as external units and are removed from the blood circulation. After the chitosan-based NPs enter in the blood system the following events occur: (1) fast formation of PC by association of NPs with plasmatic proteins; (2) rearrangement of proteins in “hard” and “soft” corona layers; (3) possible activation or inactivation on metabolic enzyme cascades; (4) induction of immune response and induction of recognition process of PC by immune cells; (5) initiation of macrophage uptake of NPs coated with PC and their elimination from the bloodstream; (6) subsequent accumulation of NPs coated with PC in some specialized cells (excluding macrophages); (7) eventually initiation of some signaling pathways and apoptosis processes [75,76,77].

Among the mechanisms that assist the entrance of NPs into the cells are pinocytosis, caveolae or clathrin assisted endocytosis and caveolae/clathrin-independent endocytosis, phagocytosis. The process of entrance of NPs in the cells depends on the properties of NPs, among them the size being the most important. It is believed that NPs smaller than 120 nm adhere to endocytic uptake, while NPs bigger than 500 nm enter in the cells by phagocytosis. Sometimes, the NPs can agglomerate and are therefore capable of being phagocytized. Moreover, the “real” size of the particle is modified due to protein adsorption [77]. Table 2 presents a selection of relevant articles that describe the fate of chitosan-based NPs and their PC.

Although for biological derived entities (e.g., fragments of viruses, bacteria, proteins) the entrance in the cell is generally governed by energy-dependent saturable endocytic pathways (like receptor-mediated endocytosis), the entrance of artificial entities (like NPs) is mostly an adsorptive type of endocytosis. For NPs with a chitosan polymer on their surface (either the NPs are made from chitosan or the NPs are only coated with chitosan) the interaction with cell membranes is mainly based on nonspecific attractive electrostatic forces [70] as the existence of a specific receptor for chitosan polymer was not yet found [34]. Nevertheless, in some specific cases, the implication of the general pathway for the formation of invaginations of plasmalemma membranes that recruit cell-surface receptors (clathrin being the most studied case) was thought responsible also for the internalization of some types of NPs. Clathrin was advocated to be involved in internalization of PLGA-based NPs and of those coated with chitosan [78], although other clathrin independent mechanisms were also considered to be involved [79]. Tahara et al. explained in detail both possible situations [34]. It was also hypothesized that some types of NPs may slide completely through the plasmalemma of eukaryotic cells using a process similar of those used by bacterial cells to cross this membrane [80].

In the research stage of chitosan-based NPs, it is necessary to label the NPs in order to track their trafficking, usually with a fluorochrome. This introduces an additional interference in the normal process of uptake and drug delivery [18].

The fate of NPs in the body depends on the type of tissues where these NPs end their journey. In the gastrointestinal tract (GI), the fate on the NPs depends on: (1) diffusion through the mucus, which may be considered as the first barrier of ingested NPs to their effective entrance in the body, (2) the way the NPs interface with the epithelium, that may be considered as the second barrier and (3) the process of translocation of NPs into the cells. These processes are governed by the diffusion of NPs through agglomerated media, consisting of mucin like proteins with gel-forming characteristics that tend to reduce the rate of diffusion of NPs. As in all diffusion cases, the velocity of NPs depends on the size of particles and on the density of the matrix. An additional factor that may influence the diffusion rate of chitosan-based NPs (or NPs coated with chitosan) into the GI tract is the negative charge of highly glycosylated extracellular proteins that form this matrix, which supports the dispersion of positively charged particles [23]. The fate or at least the residence time in the GI mucosa can be modified/controlled by tailoring the surface of NPs with functionalized groups that may interact by hydrogen bonding, hydrophobic or electrostatic interactions with mucosa proteins [18,23].

The surface charge of NPs can determine the process of entrance in the cell and their fate into the cell. Yue [18] performed a study with eight different cell lineages, including fibroblastic, epithelial, endothelial, and blood cells, revealing that positively charged NPs may travel from lysosomes to the perinuclear region, while negatively charged NPs and even neutral ones are degraded into lysosomes [18].

Another example of changing the fate of NPs is presented by Amoozgar et al. who covered polyethylene glycol (PEG) NPs with low molecular weight chitosan (LMWC) [20]. The chitosan cover (with pKa value around 6.5) promotes electrostatic interactions with negatively charged cell membranes in the weakly acidic microenvironment of tumors (pH 6.8–7.2) enhancing the internalization of NPs and drug delivery in the targeted sites. Although in the absence of positive charges of chitosan the PEG NPs can travel toward the tumor cells better than the free drug, their entrance in the tumor cells is reduced due to the hydrophilic neutral nature of PEG NPs [19].

Some studies have stated that NPs can induce cell death as consequence of disturbance of cellular, subcellular and genetic behavior, i.e., the disruption of integrity of the plasma membrane, damage of mitochondria and nucleus. The key factor in cytotoxicity of NPs seems to be the oxidant and antioxidant cellular processes initiated by NP interaction with enzymes involved in these processes. More exactly, NPs can induce the formation of reactive oxygen species (ROS). Oxidative stress is caused by an imbalance between the process of forming ROSs and the processes by which ROSs are destroyed or their effects annihilated. All organisms that obtain their metabolic energy by respiration have mechanisms of repair of destruction caused by ROS presence and mechanisms that maintain a reducing cellular environment. Imbalances of normal redox state can lead to toxic effects by producing peroxides and free radicals that affect all cell components, including proteins, lipids and nucleic acids. The main cellular substructures and metabolic pathways affected are: (1) all biological membranes (2) the flow of electrons in mitochondria and leakage from the inner mitochondrial membrane (3) calcium ion levels in the endoplasmic reticulum. The production of ROS by NPs depends on their chemical reactivity and on the NP interaction with the cellular structures involved in reparative processes [81,82].

NPs smaller than 100 nm are potentially the most dangerous because of their surface/volume ratio, relative ease of penetration into cells, and relatively high content of substances that can participate in oxidation-reduction cycles. The NPs interfere with the ability of macrophages to remove foreign particles, as phagosomes are incapable to engulf long and stiff structures. To eliminate them, the cells produce and excrete larger quantities of hydrolytic enzymes and oxygen radicals, which will install and maintain a chronic inflammation. This situation is similar with that of asbestos fibers, when chronic inflammation led to mutagenesis [83].

Beside the structure of NPs, including the possible presence of a different polymer used to coat the core of NPs, the structure of the PC greatly affects the cellular uptake and internalization processes. Covering NPs with physiological proteins can shield the charge of the plain NPs and can influence the fate of these particles in relation with different types of cells. In some cases an enhancement of cellular uptake was reported and in others an inhibition was observed [21,84,85]. For example, uptake into monocytes is enhanced by the presence of immunoglobulins and opsonins [86], while the presence of dysopsonins in the PC have an opposite effect [87]. Based on these reports one may conclude that the in vitro obtained results, when the cell uptake is studied only in buffers or in the presence of some specific proteins in PC, cannot be reproduced when the NPs are studied in body fluids. Gaspar [88] presented an example when NPs coated with transferrin have a good uptake in culture media (based on foetal bovine serum albumin), but a decrease in the overall uptake of NPs was observed when the experiments were carried out in serum. In fact, beside proteins, NPs can interact with almost all cellular components (e.g., DNA, lipids), regardless of whether this interaction takes place outside or inside of the cells. These interactions are difficult to control or predict. There are examples when the interactions of NPs with intracellular components have led to production of reactive oxygen species and/or initiation of genotoxic, inflammatory and immunological processes [89].

## 6. Applications in Drug Delivery of Chitosan-Based NPs-PC Complexes

As described in previous sections, the formation of PC changes the NPs’ properties and behavior. Controlling the behavior of the NP in the body can be done by controlling the formation of the PC. If the interactions between NP and proteins from the biological fluids are not controlled, then NPs can be toxic or dangerous, as such a nanosystem can enhance phagocytosis processes, activate enzymatic cascades in an unpredictable way, can accumulate in the body or can be expelled. In order to use NPs in medicine (e.g., for drug delivery), one should control the properties of NPs after entrance in the body, the formation of PC, the physiological response toward modified NPs [22]. Most of the times it is difficult to design/control the way a NP will interact with proteins and cells. The PC consists of tens and hundreds of proteins attached to NPs with different affinities, in different quantities and at different moments during the journey of the NP into the body. At the same time, the cells that meet the NP will have different phenotypes and surface receptor expression levels, and their behavior towards the same type of NP can be different from one individual to another. Many of these aspects were already reviewed in several papers [29,37,75,76]. Table 3 presents a selection of relevant articles that describe the type of chitosan-based NPs used to achieve the transport of a specific drug to the targeted cells.

As the NPs introduced in biological fluids interact with molecules presented in this microenvironment—chiefly with proteins—and form PCs with properties closely depending on the type of the adsorbed molecules, the surface of the NPs can be, to some extent, tailored to adsorb only those proteins that will meet some requirements (electrostatic charge, a certain level of hydrophobicity, a defined size, etc.). For example, Almalik et al. made chitosan-based NPs and particles with HA or Alg on their surfaces and compared the behavior of these three types of NPs in the presence of biological fluids. The presence of HA conducted to a reduced adsorption of proteins and to a meagre production of proinflammatory proteins. The HA present on the surface of chitosan-based NPs promote the differential adsorption of anti-inflammatory proteins ITIH4 and AGT, which were not detected on the PC of the other two types of particles. In counterpart, the PC of particles without HA, chitosan NPs and Alg-chitosan NPs selectively contained the proinflammatory protein clustrin [36].

A similar strategy of modifying the surface of the NP was applied to particles having the core formed by polyethylene glycol and, this time, the surface characteristics were modified by adding low molecular weight chitosan (LMWC), which made the resulting NPs positively charged. The reason for such alterations was to change the interaction between NPs and the target cells for the purpose of drug delivery. In the mildly acidic pH in the microenvironment of cancer cells the presence of LMWC permits the rapid uptake of NPs into cells and release of the drug due to the presence of some factors or conditions that disorganize the structure of the particle surface or hydrolyze the polymer. Amoozgar et al. present the case where fragments of 2 to 22 kDa of chitosan were released from the surface of NPs (the core made from poly(lactic-co-glycolic acid) by hydrogen peroxide digestion with subsequent release of the drug contained in the structure of the particle [20].

### 6.1. Cancer Therapy

Changing the chemical structure of NPs or at least of their surface is demanded by the targeted application and in close correlation with the fate of the transformed NP once the target tissue is located. In drug delivery—one of the most studied applications—NPs are designed to reach cancer cells without affecting normal cells. NPs having a core made from PLGA—used to load and retain drugs—were coated with LMWC in order to have a positively charged surface to the uptake into tumor cells. This ensures that benefits are provided by both polymers. PLGA’s hydrophilicity leads to a better control of adsorption and release of the drug while the positive electric charge of LMWC ensures the control of uptake by tumor cells in their mildly acidic environment [19].

In experiments on murine ascites hepatoma H22, the use of NPs to deliver doxorubicin as an antitumor agent has been proven to reduce the toxicity of the drug and to increase the survivability of the mice. In this case the chitosan-based NPs were conjugated with BSA-dextran and loaded with doxorubicin, adsorption favored at pH 7.4 by the presence of BSA on the surface of NPs [90].

Nam et al. reported the use of hydrophobically modified glycol chitosan NPs carrying 5ß-cholanic acid, an antitumor agent. These particles, having an average diameter of 359 nm and a zeta potential of 22 mV, manifested an enhanced distribution in the whole cells compared with parent hydrophilic NPs. Furthermore, the modified NPs presented better biocompatibility and lower toxicity [39].

In a recent study, chitosan-based NPs were modified by covalent linking to chitosan chain cysteine and folic acid and loaded with methotrexate, an anticancer agent. The linked molecules granted redox responsiveness and active targeting of folate receptors properties to the NPs. When used in HeLa cancer cell cultures these modified NPs presented an elevated level of reductive agents in the microenvironment of tumor cells and a controlled methotrexate release directly to this target site due to a possible overexpression of folate receptors. These properties are somehow related as in the reduced microenvironment of the cancer cells the encapsulated methotrexate was rapidly released (free methotrexate has poor water solubility and low bioavailability) [16].

SN-38—an antineoplastic drug 1000 times more active than the irinotecan from which it derives—conjugated with HA was used as shell of chitosan-based NPs decorated with MUC1 (Mucin 1, cell surface-associated) aptamer. NPs bearing MUC1 were taken up with increased efficiency in a HT29 cell culture. Unlike those lacking the drug, NPs bearing SN-38 exerted cytotoxicity through apoptosis. When both types were preincubated with bovine serum albumin that formed a PC, none of the particle types showed cytotoxicity [17].

Chitosan was added in various ratios to PLGA (poly(lactic-co-glycolic acid)) NPs to fine-tune the delivery of the carried drug. Lu et al. prepared chitosan—PLGA particles, mixing these components in various proportions by using a high-gravity rotating packed bed method. The addition of chitosan increased the size of the particles (from 132 to 172 nm), zeta potential (from -20 to 25 mV) and encapsulation efficiency (from 65% to 87%). In the meantime the drug release was slowed down from 66% to 14%, measured after 2 h. Particles with chitosan released the drug faster at pH 5.5 than 7.4 [41].

Another example of tuning the properties of NPs used as a carrier for drug delivery was presented by Aldawsari et al., who prepared PLGA NPs coated with chitosan. The carried drug was resveratrol, a well-known polyphenolic compound with many applications and anti-inflammatory, antioxidant and anti-cancer activities. Elevating the chitosan concentration led to an increase in particle size (up to 340 nm), zeta potential (from negative to positive, up to 26 mV), entrapment efficiency (up to 75%), aid in the stability of the drug in NPs. Compared with free resveratrol, the PLGA chitosan-coated NPs carrying this drug presented higher cytotoxicity and apoptotic activities [42].

Similar NPs, with PLGA core and coated with chitosan, but obtained by another technique (water-oil-water emulsion of poly(DL-lactic-co-glycolic acid) solvent evaporation, followed by surface-modified by adsorption of chitosan) were used as a drug carrier for pulmonary administration (human lung adenocarcinoma cells A549). Compared with NPs made only from PLGA, the particles coated with chitosan manifested low cytotoxicity and were a better carrier of the drug, the uptake was higher and the release of the drug was slower. At least in the case of A549 cells, both types of NPs were taken up in an energy-dependent manner by a clathrin-mediated endocytic process [34].

Aiming to enhance the biocompatibility of curcumin towards A549 cells, the drug was loaded on NPs made from biotin-chitosan-dithiodipropionic acid-curcumin. The addition to these NPs of a type of phycocyanin-functionalized corona gives them the potential to avoid protein adsorption in blood circulation. The release of curcumin from the redox responsive shells of the NPs was sensitive to a high concentration of glutathione. The enhanced intracellular uptake of curcumin increased the inhibitory activity on the proliferation of A549 cells [45].

Reduction-responsive HA–chitosan–lipoic acid nanoparticles (HACSLA-NPs) were designed and synthesized for effective treatment of breast cancer. The nanodelivery system was used to target CD44-overexpressing cells and release of reduction-triggered 17-methyltestosterone (MT) for systemic delivery. In vitro experiments showed that MT/HACSLA-NPs presented a fast drug release in the presence of glutathione, while the absence of glutathione led to a sustained MT release. HACSLA-NPs showed higher cellular uptake via CD44 receptors, rapid release of the drug inside the cells, and increased cytotoxicity against BT-20 cell lines as opposed to MCF-7 cell lines. Both MT/CSLA-NPs and MT/HACSLA-NPs were highly efficient in targeting breast cancer cell lines, but MT/HACSLA-NPs presented higher selectivity, cytotoxicity and apoptotic effect on BT-20 cells compared to MT/CSLA-NPs [47].

### 6.2. DNA Transfection

An additional application of chitosan-based NPs is the use of these particles as vectors to transfect the cells. Douglas et al. have shown the transfection efficiency of fluorescently labeled alginate-chitosan NPs complexes, at least in the case of some cell lines (293T and COS7) that possess the clathrin-mediated endocytosis pathway [40].

Robles-Planells et al. evaluated the effects of the expression of a fusogenic protein from the infectious salmon anemia virus (ISAV-F) on a murine B16 melanoma model, in vitro as well as in vivo, using chitosan NPs as transfection vectors. The transfection of B16 murine tumor cells with ISAV-F-loaded chitosan NPs allowed the expression of the ISAV-F protein and lowered cell viability. The in vivo transfection slightly delayed tumor growth. Expression of ISAV fusion protein using chitosan NPs induced fusion of melanoma cells and slight in vivo antitumoral effect in comparison to chitosan treatment [35].

### 6.3. Insulin Delivery

Another interesting application of chitosan-based NPs is their use as a core that was covered with pluronic F127 and finally used to transport insulin. Pluronic F127 is composed of triblock PEO–PPO–PEO copolymers of poly(ethylene oxide) (PEO) and poly(propylene oxide) (PPO). In an aqueous environment, these block copolymers self-assemble into micelles with a hydrophobic PPO oriented toward the interior and a hydrophilic PEO oriented toward the water. In a study of the applications of these special NPs insulin was nested inside, where it was protected by enzymatic degradation—which drugs carried by orally administrated NPs may suffer on their way to the target tissue. The cellular level of insulin after its administration into the chitosan-pluronic particles was 36-fold higher compared with free insulin and 10-fold higher compared with particles made only from chitosan. Several other properties have also been improved by carrying insulin with chitosan-pluronic particles: efficiency of mucosal penetration, cellular internalization of insulin in mucus-secreting E12 cells, permeation of insulin across the ileum epithelia, hypoglycemic effects in diabetic rats, and so on [15].

### 6.4. Delivery of Antibiotics

Another well documented application of NPs for drug delivery is the use of NPs for antibiotic delivery as near as possible to the target cells. For this, the core of NPs or at least the coverage of the core is tailored to interact in a predictive matter with the most abundant proteins located in the same place as the target cells. The adsorption and delivery of the drug was then studied mimicking the conditions from the near environment of the target cells. For example, Niaz et al. [43] reported the realization of chitosan-based NPs, having on the surface polylysine polymer with alternative corona layers (combination of NPs bearing on their surface polylysine, BSA, plain chitosan, chitosan-shell on BSA-core or BSA-shell on chitosan-core) to be used against multidrug resistant gastric *H. pylori*, based on the antimicrobial properties of the polylysine peptide. NPs having the core of BSA and surface of chitosan have been proven to present the best antimicrobial activity, mucoadhesion and to better release the polylysine peptide, at least in simulated gastric conditions. Considering the special conditions of the gastrointestinal cavity, the “reversed” NPs proposed by Niaz, i.e., with the core of protein (BSA), covered with chitosan and on top with polylysine adsorbed on chitosan surface, although atypical, seems to give good results (increased mucoadhesivity, enhanced bioavailability). There are published results based on typical layer disposition, i.e., core of chitosan-base (more precisely thiolated chitosan/PMLA) having adsorbed antibiotics (amoxicillin) on the surface of NPs used for the delivery of the drug into the gastrointestinal cavity to treat *H. pylori* [43,91].

### 6.5. Delivery of Antiarrhythmic Drugs

Chitosan was used in an NP drug delivery system for the controlled release of hydrophobic amiodarone (AMD) along with the cyclic oligosaccharide β-cyclodextrin, which increases the solubility of hydrophobic molecules in water. Amiodarone-loaded chitosan nanoparticles were prepared using the ionic gelation method with a reaction yield of 11–15%, an encapsulation efficiency of 33–36% and a loading capacity of 8–9%. In this in vitro release study, all of the AMD was released after 14 days, 38% at the end of day 1 and 50% at the end of day 5. The authors concluded that the AMD-loaded chitosan NPs might be used for long-term treatment with AMD and could be a model for controlled delivery of other antiarrhythmic drugs [46].

## 7. Conclusions

Depending on the application of the NPs, their core, surface and to a small extent, the composition of the PC in biological media may be tailored. For most drug-carrying NPs, the size and the particle charge are the main parameters that have to be tuned to obtain a good uptake of the carried drug to the target cells.

The type of the target cells is important due to the differences between the proteins, polysaccharides and other types of molecules carrying electric charges located in plasma membranes of the target cells and due to different types of endocytosis mechanisms. The smaller particles travel faster through mucus than larger ones due to diffusion. When enterocytes are the target cells, the smaller the size of NPs, the quicker is their transport through the intestinal mucosa.

The PC can be formed by adsorption on the NPs surface of proteins (and other types of molecules) found in the biological fluids through which the drug-carrying particles travel towards the target cell. The type and the concentration of proteins in the PC are constantly changing depending not only on the size, composition and charge of NPs but also on the duration of the trip.

Chitosan-based NPs are mainly used for the delivery of anticancer drugs to tumor cells. Other uses include delivering antibiotics closer to the target tissue, insulin transport, DNA transfection and others.

## Figures and Tables

**Table 1:** Physico-chemical characterizations of chitosan-based NPs influencing PC formation in different cell types.

Reference	Nanoparticle Types	Method of NPs Synthesis	Average Size (nm)	Polydispersity Index (PDI)	Zeta Potential (mV)	Cell Type
Nam 2009	Hydrophobically modified glycol chitosan NPs	Partial derivatization of the free amino groups of glycol chitosan (GC) with 5β-cholanic acid	359 nm	0.009	+22	Human HeLa cells
[39]						
Mazzotta 2020 [16]	Folic-thiolated chitosan (FTC1) NPs	Ionotropic gelation (65.87% disulfide bond)	364.2 ± 3.1	0.167	28.3 ± 1.04	Human cervix adenocarcinoma (HeLa) cell culture
	FTC2 NPs	Ionotropic gelation	202.4 ± 5.8	0.254	35.9 ± 1 0.36	
		(92.29% disulfide bond)				
	FTC3 NPs	Ionotropic gelation	234.7 ± 7.9	0.234	24.9 ± 1.4	
		(70.71% disulfide bond)				
	Chitosan	Ionotropic gelation	378.4 ± 7.4	0.318	+30.7 ± 3.35	
	Methotrexate-loaded folic-thiolated chitosan (FTC1-MTX) NPs	Ionotropic gelation with addition of MTX alkaline solution	363.9 ± 3.3	0.154	+26.3 ± 1.18	
	FTC2-MTX NPs		258.3 ± 4.9	0.153	+28.9 ± 0.47	
	FTC3-MTX NPs		302.0 ± 9.3	0.25	+26.8 ± 1.89	
	Chitosan-MTX		364.1 ± 2.5	0.273	+26.7 ± 0.99	
Douglas 2008	Alginate–chitosan NPs	Spontaneous complex coacervation	157	-	+32	Human 293T Monkey COS-7 Hamster CHO
[40]						
Almalik 2017 [36]	Chitosan NPs	Ionotropic gelation method	120 ± 20	0.30 ± 0.03	+34 ± 5	Cells bearing Cluster of Differentiation (CD44) receptors
	HA-Chitosan NPs	Ionotropic gelation with addition of acetate buffer containing hyaluronic acid (HA)	270 ± 27	0.22 ± 0.04	−32 ± 5	
	Alg-Chitosan NPs	Ionotropic gelation with addition of acetate buffer containing alginate (Alg)	790 ± 70	0.46 ± 0.05	−72 ± 8	
Amoozgar 2012 [20]	PLGA NP (pH 7.4)	Low molecular weight chitosan (LMWC) produced by hydrogen peroxide digestion and covalently conjugated with poly(lactic-co-glycolic acid) (PLGA) Chitosan with a MW of 15 kDa (LMWC15k) was added to the continuous phase to form a physical coating on PLGA NP (PLGA/LMWC15k NP).	177.5 ± 40.2	0.15 ± 0.1	−11.1 ± 3.1	SKOV-3 and NCI/ADR-RES cancer cells
	PLGA/LMWC_15k_ NP (pH 7.4)		175 ± 12.0	0.25 ± 0.07	−12.0 ± 2.0	
	PLGA/LMWC2_−4k_ NP (pH 7.4)		176.0 ± 45.2	0.23 ± 0.09	−6.0 ± 2.3	
	PLGA/LMWC4_−6.5k_ NP (pH 7.4)		191.6 ± 34.1	0.18 ± 0.01	−4.4 ± 1.2	
	PLGA/LMWC12_−22k_ NP (pH 7.4)		480.0 ± 21.0	0.17 ± 0.04	−9.3 ± 4.0	
	PLGA NP (pH 6.2)		191.6 ± 43.2	0.09 ± 0.01	−14.6 ± 4.3	
	PLGA/LMWC_15k_ NP (pH 6.2)		184.1 ± 11.5	0.29 ± 0.02	−10.1 ± 1.8	
	PLGA/LMWC2_−4k_ NP (pH 6.2)		183.3 ± 47.4	0.10 ± 0.01	+3.3 ± 1.4	
	PLGA/LMWC4_−6.5k_ NP (pH 6.2)		198.8 ± 28.9	0.13 ± 0.02	+5.5 ± 1.9	
	PLGA/LMWC12_−22k_ NP (pH 6.2)		404.1 ± 31.3	0.12 ± 0.06	+14.9 ± 0.9	
Lu 2019 [41]	PLGA	Nanoprecipitation using high-gravity rotating packed bed reactor	132.8 ± 1.5	0.155 ± 0.03	−20.8 ± 1.1	MDA-MB-231 tumor cells
	Chitosan/PLGA (w/w) = 0.2		140.5 ± 2.4	0.104 ± 0.02	10.1 ± 0.9	
	Chitosan/PLGA (w/w) = 0.4		154.2 ± 2.6	0.122 ± 0.04	21.5 ± 0.5	
	Chitosan/PLGA (*w*/*w*) = 0.8		172.7 ± 3.2	0.144 ± 0.06	25.6 ± 0.6	
Aldawsari 2020 [42]	PLGA NPs	Single emulsion-sonication method	271.63 ± 13.81	0.123 ± 0.08	−2.55 ± 0.28	Human non-small cell lung carcinoma (NSCLC) cell line (H1299)
	Resveratrol-PLGA NPs		286.13 ± 11.64	0.351 ± 0.02	−4.16 ± 1.13	
	Chitosan-coated PLGA NPs		349.10 ± 17.92	0.358 ± 0.01	29.3 ± 0.60	
	Chitosan-coated Resveratrol-PLGA NPs		341.56 ± 7.90	0.117 ± 0.01	26.88 ± 2.69	
Li 2013 [15]	core shell corona nanolipoparticles (CSC)	Hydration of a F127-lipid film (prepared by drying a chloroform solution containing egg phosphatidylcholine with F127) with NC suspension to form core shell structure	195.3 ± 32.9	0.151 ± 0.048	−4.3 ± 5.4	Human mucus-secreting HT29-MTX-E12 cells
	core shell nanolipoparticles without hydrophilic corona (CS)	Encapsulation of chitosan NPs in a pure lipid vesicle without Pluronic F127.	210.5 ± 45.3	0.311 ± 0.075	+36.6± 4.5	
	Chitosan NPs	Ionotropic gelation	202.8 ± 22.9	0.175 ± 0.069	−7.1 ± 3.2	
Niaz 2019 [43]	Bovine serum albumin nano delivery system (BSA-NDS)	Ionotropic complexation and layer by layer assembly	125.6 ± 1.0	0.206	−22.3 ± 4	*H. pylori* culture
	ε-poly-L-lysine BSA-NDS		184 ± 15	0.329	−16.7 ± 2	
	Chitosan-shell on BSA-core (C(B)NDS)		223 ± 1.7	0.269	27.1 ± 1.6	
	ε-poly-L-lysine-C(B)NDS		372 ± 2.0	0.351	20.4 ± 1.9	
	Chitosan NDS		145 ± 2.2	0.291	33.9 ± 5.4	
	ε-poly-L-lysine (ε-PL)-Chitosan-NDS		164 ± 4.0	0.318	35.9 ± 2	
	BSA-shell on Chitosan-core (B(C)NDS)		191 ± 2.6	0.21	−31 ± 2.5	
	ε-poly-L-lysine-B(C)-NDS		231 ± 3.0	0.269	−15.4 ± 1.3	
Varnamkhasti. 2015 [17]	Aptamer modified NPs	Ionotropic gelation.	129 ± 3.2	0.31 ± 0.021	14 ± 1.2	HT-29 (human colon cancer cell line), MUC1 positive cell line
		SN-38 conjugated to hyaluronic acid (HA) used as the shell of chitosan NPs, further modified with MUC1 aptamer				
	Unmodified NPs	Ionotropic gelation.	126 ± 2.1	0.27 ± 0.032	14.8 ± 1.5	
		SN-38 conjugated to HA used as the shell of chitosan NPs				
Kim 2008 [44]	Chitosan uncoated PLGA/PVA NPs	Double emulsion-solvent evaporation technique using PLGA and an aqueous polyvinyl alcohol (PVA) solution	202.2 ± 3.2	0.13 ± 0.02	−30.1 ± 0.6	H157 human lung cancer cells
	chitosan (0.2 mg/mL) coated PLGA/PVA NPs		210.1 ± 4.1	0.16 ± 0.05	11 ± 0.8	
	chitosan (0.5 mg/mL) coated PLGA/PVA NPs		212.0 ± 3.9	0.18 ± 0.06	26 ± 1.2	
	chitosan (1.0 mg/mL) coated PLGA/PVA NPs		212.2 ± 2.9	0.19 ± 0.08	26 ± 1.2	
Tahara 2009 [34]	Non-PLGA 1000	Water–oil–water emulsion solvent evaporation method	939 ± 23.9	-	−30.8 ± 3.8	human lung adenocarcinoma cells (A549)
	Non-PLGA 400		410.0 ± 26.3		−33.9 ± 2.2	
	Non-PLGA 200		410.0 ± 26.3		−28.5 ± 1.1	
	Chitosan-PLGA 1000		1109.1 ± 20.7		−3.8 ± 0.6	
	Chitosan-PLGA 400		475.2 ± 16.0		−4.6 ± 1.1	
	Chitosan-PLGA 200		248.9 ± 4.1		−3.1 ± 0.7	
Yue 2011 [18]	negatively charged NPs (N-NPs)	Initial fabrication of carboxymethyl chitosan (CMC) NPs by the SPG membrane emulsification technique	215.70 ± 2.91	0.054 ± 0.0051	−45.84 ± 2.18	Eight cell lines: epithelial cells A549 and HKC fibroblastic cells MRC-5 and CCC-HSF-1 endothelial cells HUVEC and CRL-2472blood cells UT-7 and K562
	neutrally charged NPs (M-NPs)	Subsequent deposition of N-NPs with a layer of chitosan	214.27 ± 1.36	0.059 ± 0.0038	0.51 ±1.31	
	positively charged NPs (P-NPs),	Subsequent deposition on N-NPs with a layer of N-[(2-hydroxy-3-trimethylammonium) propyl] chitosan chloride (HTCC)	216.12 ± 3.57	0.052 ± 0.0042	39.25 ±2.68	
Cheng 2019 [45]	CUR-BCSCs	Curcumin-loaded (CUR) biotin-chitosan oligosaccharide-dithiodipropionic acid-curcumin (BCSC) NPs (CUR-BCSCs) prepared through the self-assembly method from BCSC and CUR solution	97.8 ± 4.2	0.181 ± 0.014	21.57 ± 0.53	A549 cells
	CUR-BCSC-Phycocyanin	CUR-BCSCs with addition of an aqueous solution of phycocyanin	160.3 ± 9.0	0.114 ± 0.024	12.90 ± 1.93	
Buyuk 2020 [46]	NP1 (3.0 mg/mL β-cyclodextrin (β-CD))	Ionic gelation followed by ultrasonication	172.5 ± 8.2	0.39 ± 0.048	+27.2	-
	NP2 (3.5 mg/mL β-CD)		228.3 ± 9.7	0.48 ± 0.033	+26.0	
	NP3 (3.0 mg/mL β-CD, 0.5 mg/mL amiodarone (AMD))		296.8 ± 4.1	0.41 ± 0.023	+29.4	
	NP4 (3.5 mg/mL β-CD, 0.5 mg/mL AMD)		372.8 ± 11.53	0.44 ± 0.036	+29.7	
Robles-Planells 2020 [35]	N/P4 (ratio of -NH_2_ group of chitosan versus -PO_4_^2−^ group of pDNA)	Coacervation	32.7	Monodisperse	−1.22	B16 tumor cells
	N/P20		68.1		7.15	
	N/P28		68.1		2.08	
					and 31.8	
	N/P40		78.8		−2.81 and 23.2	
Rezaei 2020 [47]	Chitosan–lipoic acid nanoparticles (CSLA-NPs)	Amidation reaction	240 ± 0.056	0.369 ± 0.056	+26	CD44-overexpressing cells
	Hyaluronic acid Chitosan–lipoic acid nanoparticles (HACSLA-NPs)		280 ± 0.045	0.327 ± 0.002	+19	
Ciro 2020 [48]	DCH-PAM-2Na (Chitosan NPs modified with sodium salt of poly(maleic acid-alt-ethylene))	Polyelectrolyte complexation assisted by high-intensity sonication	198.7	0.182	+20.0	-
	DCH-PAM-2K (Chitosan NPs modified with potassium salt of poly(maleic acid-alt-ethylene))		172.9	0.181	+20.6	
	DCH-PAM-18Na		156.5	0.396	+31.2	
	(Chitosan NPs modified with sodium salt of poly(maleic acid-alt-octadecene))					
	DCH-PAM-18K		217.0	0.387	+30.5	
	(Chitosan NPs modified with potassium salt of poly(maleic acid-alt-octadecene))					
	MTX-DCH-PAM-2Na		172.5	0.232	32.1	
	MTX-DCH-PAM-2K		151.6	0.183	33.3	
	MTX-DCH-PAM-18Na		166.1	0.344	36.6	
	MTX-DCH-PAM-18K		145.2	0.300	39.6	

**Table 2:** The fate of chitosan-based NPs and their PC in various biological fluids and sites.

Reference	Type of Chitosan-Based NPs	Chemical Composition of NP	Fate of Chitosan-Based NP and Their PC
Nam 2009 [39]	Functionalized chitosan-based NP	Hydrophobically modified glycol chitosan (HGC) NP	Some of the HGC NPs were localized in the late endosomes and lysosomes, and a fewer amount was detected in the endoplasmic reticulum region.
			The HCG NPs exhibited a fast cellular uptake through various routes.
Mazzotta 2020 [16]	Functionalized chitosan-based NP	Folic-thiolated chitosan nanoparticles (FTC1) NP	Folic acid-decorated redox-responsive NPs were able to enhance the intracellular release and to target drug selectivity in tumor cells. FTC-NPs showed a better inhibition effect on HeLa cancer cell proliferation compared to non-target chitosan-based NPs used as control, demonstrating a better uptake of the NP compared to control. The selective cellular uptake of FTC-NPs occurred via folate receptors.
		FTC2 NP	
		FTC3 NP	
	Plain chitosan NP	Chitosan NP	
	Functionalized chitosan-based NP	Methotrexate-loaded folic-thiolated chitosan (FTC1-MTX) NP	
		FTC2-MTX NP	
		FTC3-MTX NP	
	Plain chitosan NP	Chitosan-MTX NP	
Douglas (2008) [40]	Functionalized chitosan-based NP	Alginate–chitosan NP	Human 293T cells, clathrin-mediated endocytosis:
			Following internalization, complexes (alginate-chitosan NPs) are trafficked to late endosomes and/or lysosomes, where acidification is countered by the proton-sponge pH buffering capacity of chitosan within the complexes. This effect results in endosomal rupture, escape of the complexes, and ultimately leads to transfection.
			Monkey COS-7 cells, clathrin-mediated endocytosis:
			Complexes entering through the clathrin-dependent process are presumed to be trafficked similarly as in human 293T cells, leading to transfection.
			Monkey COS-7 cells, caveolin-mediated endocytosis:
			These complexes are entrapped in caveosomes but are not trafficked to the endo-lysosomal pathway. Since these vesicles do not undergo acidification, remains no mechanism for the complexes to escape; they consequently remain entrapped in vesicles where they cannot mediate transfection.
			Hamster CHO cells, caveolin-mediated endocytosis:
			Caveolin-mediated endosomes and not lysosomes
Almalik 2017 [36]	Plain chitosan NP	Chitosan NPs	CS and Alg-CS NPs selectively adsorbed a proinflammatory protein (Clusterin) that was not found on the surfaces of HA-CS NPs. -HA-CS NPs differentially adsorbed two unique anti-inflammatory proteins (ITIH4 and AGP), which were absent from the PC of both controls (CS and Alg-CS NPs)
	Functionalized chitosan-based NP	HA-Chitosan NPs	
		Alg-Chitosan NPs	
Amoozgar 2012 [20]	Functionalized chitosan-based NP	PLGA NP (pH 7.4 and pH 6.2)	The hydrophilic LMWC layer reduced opsonization and phagocytic uptake.
		PLGA-LMWC_2−4k_ NP (pH 7.4 and pH 6.2))	PLGA*-LMWC_2−4k_ NP effectively avoided uptake by J774A.1macrophages, whereas PLGA* NP was readily taken up by them. This result was obtained at pH 7.4, where both NPs were negatively charged; therefore, contribution of electrostatic interactions with cells to the cellular uptake was minimal for both NPs.
		PLGA-LMWC_4−6.5k_ NP (pH 7.4 and pH 6.2))	The pH responsiveness of surface charges of PLGA-LMWC NPs translated to differential NP-cell interactions at the pH 7.4 and pH 6.2.
		PLGA-LMWC_12−22k_ NP (pH 7.4 and pH 6.2)	If the cellular uptake experiments were performed in pH 7.4, the difference might be attributable to relatively high MWs of the chitosans, which enhanced nonelectrostatic interactions such as hydrogen bonding and hydrophobic interactions between the chitosan layer and cell membranes.
Lu 2019 [41]	Functionalized chitosan-based NP	Chitosan-modified PLGA NPs	The cellular uptake and cytotoxicity of chitosan-modified PLGA NPs was higher compared with PLGA NPs in MDA-MB-231 cells
Abouelmagd 2015 [19]	Functionalized chitosan-based NP	(poly(lactic-co-glycolic) acid—low molecular weight chitosan (PLGA-pD-LMWC) NPs	While PLGA-pD-LMWC NPs did not interact with cells at normal physiological pH, they were able to establish interactions with cells at pH < 6.5 and get internalized into the cells without being trafficked into the acidic organelles. The LMWC layer did not completely prevent protein binding to the NPs incubated in serum solution but reduced phagocytic uptake.
Li 2013 [15]	Functionalized chitosan-based NP	Core shell corona nanolipoparticles (CSC)	The increased level of cellular insulin uptake observed with CSC in E12 cells showed 10-fold higher uptake compared to NC. The unmodified CS also enhanced insulin transport to a less extent as compared to CSC. When reaching the small intestine, NC were mostly immobilized in the mucin network, but CSC could penetrate through the mucus and thus more insulin could reach the epithelium surface and be transported across the intestinal epithelium via the paracellular pathway, transcytosis or receptor-mediated transcytosis.
		Core shell nanolipoparticles without hydrophilic corona (CS)	
	Plain chitosan NP	chitosan nanoparticles (NC)	
Niaz 2019 [43]	Functionalized chitosan-based NP	Bovine serum albumin nano delivery system (BSA-NDS)	CS corona dissociate once interacted with the gastric mucosa. As chitosan lose its charge and become deprotonated at mucosal pH, this could release the core BSA-NDS with remaining encapsulated protein, which can penetrate deep into mucus membrane.
		ε-poly-L-lysine BSA-NDS	
		Chitosan-shell on BSA-core (C(B)NDS)	
		ε-poly-L-lysine-C(B)NDS	
	Plain chitosan NP	Chitosan NDS	
	Functionalized chitosan-based NP	ε-poly-L-lysine (ε-PL)-Chitosan-NDS	
		BSA-shell on Chitosan-core (B(C)NDS)	
		ε-poly-L-lysine-B(C)-NDS	
Varnamkhasti 2015 [17]	Functionalized chitosan-based NP	Aptamer modified NPs (SN-38 conjugated to hyaluronic acid (HA), further modified with MUC1 aptamer)	SN-38 is attached via an esteric bond to HA with the help of glycine as a linker. Due to the sensitivity of esteric bonds to lower pHs, this bond is easily cleaved leading to higher release of the drug. The overall cumulative release of SN-38 at the lower pH present in cancer cells (pH 5.2) is nearly twice the release at pH 7.4. Uptake of the aptamer-modified NPs by HT29 was twice higher than the unmodified nanoparticles. The PC induced a reduction in the uptake of the targeted NPs.
		Unmodified NPs (SN-38 conjugated to HA)	
Kim 2008 [44]	Non-chitosan NP	Chitosan uncoated PLGA/PVA NPs	The uptake of chitosan coated NPs was much higher than that of the uncoated NPs. The internalization of cationic chitosan NPs occurs predominantly by adsorptive endocytosis.
	Functionalized chitosan-based NP	Chitosan (0.2 mg/mL) coated PLGA/PVA NPs	
		Chitosan (0.5 mg/mL) coated PLGA/PVA NPs	
		Chitosan (1.0 mg/mL) coated PLGA/PVA NPs	
Tahara 2009 [34]	Non-chitosan NP	Non-PLGA 1000	Cellular uptake of PLGA nanosystems increased with decreasing diameter to the submicron level and with chitosan-mediated surface modification. Cellular uptake of PLGA NS was energy dependent, as shown by a reduction in uptake at lower incubation temperatures and in hypertonic growth medium used as an inhibitor of clathrin-coated pit endocytosis.
		Non-PLGA 400	
		Non-PLGA 200	
	Functionalized chitosan-based NP	Chitosan-PLGA 1000	Particle size significantly affected cellular uptake in A549 cells; only submicron-sized (200-nm) particles were taken up efficiently, and not the large-sized microparticles (1µm). Nanosystems with a size of 200 nm showed ~2.5-fold greater uptake than those with a size of 1µm by the A549 cell line. CS-PLGA NSs were taken up by A549 cells in an energy dependent manner, suggesting a clathrin-mediated endocytic process.
		Chitosan-PLGA 400	
		Chitosan-PLGA 200	
Yue 2011 [18]	Functionalized chitosan-based NP	Negatively charged NPs	The cellular uptake rate and amount are both positively correlated with the surface charge in all cell lines. Subsequent intracellular trafficking indicates that some of positively charged NPs could escape from lysosome after being internalized and exhibit perinuclear localization, whereas the negatively and neutrally charged NPs prefer to colocalize with lysosome.
		Neutrally charged NPs	
		Positively charged NPs (P-NPs),	
Cheng 2019 [45]	Functionalized chitosan-based NP	CUR-BCSCs (curcumin (CUR)-loaded biotin-chitosan oligosaccharide-dithiodipropionic acid-curcumin (BCSC) NPs)	Both CUR-BCSCs and CUR-BCSC@PCs were absorbed in A549 cell lines, and the uptake efficiency was time-dependent. Cellular uptake took place through caveolae-mediated endocytosis. The cell uptake rate of CUR-BCSC@PCs was high.
		CUR-BCSC@PCs (phycocyanin (PC)-functionalized and curcumin (CUR)-loaded biotin-chitosan oligosaccharide-dithiodipropionic acid-curcumin (BCSC) NPs)	
Buyuk 2020 [46]	Functionalized chitosan-based NP	NP3 (3.0 mg/mL β-CD,	Amiodarone encapsulated in NPs was completely released at the end of 14 days. About 38 % was released at the end of day 1, 44% released at the end of day 3, 50% released at the end of day 5 followed slow release.
		0.5 mg/mL AMD)	
		NP4 (3.5 mg/mL β-CD,	
		0.5 mg/mL AMD)	
		N/P20	
		N/P28	
		N/P40	
Rezaei 2020 [47]	Functionalized chitosan-based NP	Chitosan–lipoic acid NPs (CSLA-NPs)	In CD44 negative MCF-7 cell lines, both NPs can only be internalized via endocytosis. 17α-Methyltestosterone (MT)-loaded HACSLA-NPs showed higher cellular internalization via CD44 receptors than CSLA-NPs. An investigation of the cellular responses of Michigan Cancer Foundation-7 (MCF-7) and breast cancer (BT-20) cell lines to unloaded and MT-loaded NPs at varying doses showed that MT-loaded NPs would damage the plasma and mitochondrial membranes, which can be attributed to LDH release into the extracellular medium.
		Hyaluronic acid Chitosan–lipoic acid NPs (HACSLA-NPs)	
Ciro 2020 [48]	Functionalized chitosan-based NP	DCH-PAM-2Na	These chitosan-polyanion NPs modified the MTX release by an anomalous mechanism, where the NPs formed with PAM-2 polymer led to a release mechanism controlled by diffusion and relaxation, whereas the NPs formed with PAM-18 led to a mainly diffusion-controlled release mechanism.
		DCH-PAM-2K	
		DCH-PAM-18Na	
		DCH-PAM-18K	
		MTX-DCH-PAM-2Na	
		MTX-DCH-PAM-2K	
		MTX-DCH-PAM-18Na	
		MTX-DCH-PAM-18K	

**Table 3:** Applications of NPs coated with specific corona to achieve particular goal.

Reference	Chemical Composition of NP	Purpose	Goal Achievement
Nam 2009 [39]	Hydrophobically modified glycol chitosan NPs	Tumor targeting	The HGC NPs possess tunable physicochemical properties, low toxicity, biocompatibility are promising versatile macromolecular carriers for the intracellular delivery of therapeutic agents.
Mazzotta 2020 [16]	Folic-thiolated chitosan (FTC1) NPs	Tumor targeting	The designed NPs provide an attractive strategy and potential platform for efficient intracellular anticancer drug delivery.
	FTC2 NPs		
	FTC3 NPs		
	Chitosan		
	Methotrexate-loaded folic-thiolated chitosan (FTC1-MTX) NPs		
	FTC2-MTX NPs		
	FTC3-MTX NPs		
	Chitosan-MTX NPs		
Douglas (2008) [40]	Alginate–chitosan nanoparticle	DNA transfection	Alginate-chitosan NPs were used as non-viral vectors to transfect 293T, COS7, and CHO cells and to observe the cellular interactions and internalization mechanisms of the complexes in each cell line. They mediate transfection in 293T and COS7 cells, but did not lead to transfection in CHO cells.
Almalik 2017 [36]	Chitosan NPs	Control	The composition of the PC formed around the studied NPs was investigated with the goal of further researching nano drug delivery systems.
	HA-Chitosan NPs	Study of the composition of the PC	
	Alg-Chitosan NPs	Control	
Abouelmagd 2015 [19]	poly(lactic-co-glycolic) acid - low molecular weight chitosan PLGA-pD-LMWC NPs	Tumor-specific drug delivery	The PLGApD-LMWC NPs provided pH-sensitive surface layer, which enabled acid-specific NP–cell interaction and enhanced drug delivery to cells in the weakly acidic environment.
Amoozgar 2012 [20]	PLGA NP (pH 7.4 and pH 6.2.)	Tumor-Specific Drug Delivery (paclitaxel)	PLGA NPs had negative charges irrespective of pH, due to carboxyl termini exposed on the surface, and did not have significant interactions with cancer cells at both pHs 7.4 and 6.2.
	PLGA-LMWC_2−4k_ NP (pH 7.4)		There was a lack of cellular interactions of PLGA-LMWC NPs at pH 7.4.
	PLGA-LMWC_4−6.5k_ NP (pH 7.4)		
	PLGA-LMWC_12−22k_ NP (pH 7.4)		
	PLGA-LMWC_2−4k_ NP (pH 6.2)		The ability of PLGA-LMWC NP to cationize the surface at pH 6.2 and establish interactions with cancer cells makes them attractive in drug delivery to acidic tumors. The ability of cationized PLGA-LMWC_2−4k_ NP to deliver drugs through short-term exposure should allow them to serve as an effective drug delivery system to tumors
	PLGA-LMWC_4−6.5k_ NP (pH 6.2)		Considering the LMWC content, hydrophilicity of the LMWC coating, particle size, and ability to interact with cells at acidic pH, PLGA-LMWC_4−6.5k_ NP should be most appropriate for drug delivery to tumors.
	PLGA-LMWC_12−22k_ NP (pH 6.2)		Due to the large particle size, PLGA-LMWC_11−22k_ NP was excluded from the cell studies.
Lu 2019 [41]	PLGA NPs	Tumor-Specific Drug Delivery (paclitaxel)	Chitosan-modified, paclitaxel-loaded PLGA NPs exhibited sustained drug release and enhanced drug toxicity, suggesting that they can be used as carriers of anticancer drugs
Aldawsari 2020 [42]	Chitosan-coated resveratrol PLGA NPs	Tumor-Specific Drug Delivery (resveratrol)	Chitosan coating improved the stability of resveratrol-loaded PLGA NPs. Chitosan-coated NPs showed greater cytotoxicity and higher antioxidant and apoptotic activities compared to free resveratrol. Therefore, chitosan coated PLGA NPs could be a potential nanocarrier of resveratrol to increase drug solubility, entrapment, sustain release, stability and therapeutic use.
Li 2013 [15]	Core shell corona nanolipoparticles (CSC)	Intestinal mucosa permeability following oral insulin delivery	CSC have been found to improve insulin transport through E12 cells as compared to insulin solution and naked NC. In addition to their ability to enhance mucus penetration, CSC may also enhance cellular uptake of insulin by surface modification of the nanolipoparticles with F127 polymers. CSC exhibited improved stability in the GI tract, enhanced mucus penetration, and membrane transport, leading to significantly more potent and prolonged pharmacological efficacies. CSC exhibited stronger hypoglycemic effects than CS and CN.
	Core shell nanolipoparticles without hydrophilic corona (CS)		The concentration of insulin delivered by CS in the mucus layer was similar to that delivered by the CSC, but far less insulin was observed inside E12 cells in the case of CS compared to CSC.
	Chitosan NPs (CN)		Chitosan NPs increased the amount of insulin trapped in mucus. The ability of CN to enhance cellular uptake and cellular transport was less significant, they were unable to reach the epithelial surface and so failed to improve the absorption of encapsulated proteins. Compared to a plain insulin solution, which failed to reduce the blood glucose level, CN slightly reduced the blood glucose level.
Niaz 2019 [43]	Bovine serum albumin nano delivery system (BSA-NDS)	Improve the stability and controlled release of nano-medicines for gastric delivery	BSA-core having chitosan corona demonstrated better antimicrobial activity, mucoadhesion and controlled release of ε-PL in simulated gastric conditions. Conversely, NDS having PC exhibited better stability and antibiofilm activity against gastric *H. pylori*. At near neutral pH (6.8), BSA based NDS demonstrated better controlled release than CS based NDS. Whereas at acidic pH (1.2 and 3), NDS having CS corona offer better release of encapsulated peptides.
	ε-poly-l-lysine BSA-NDS		
	Chitosan-shell on BSA-core (C(B)NDS)		
	ε-poly-l-lysine-C(B)NDS		
	Chitosan NDS		
	ε-poly-l-lysine (ε-PL)-Chitosan-NDS		
	BSA-shell on chitosan-core (B(C)NDS)		
	ε-poly-l-lysine-B(C)-NDS		
Varnamkhasti 2015 [17]	Aptamer modified NPs (SN-38 conjugated to hyaluronic acid (HA), further modified with MUC1 aptamer)	Targeted delivery of SN-38 (an active metabolite of camptothecin) in HT-29 cancer cells	MUC1 aptamer is an effective targeting agent for increasing the cytotoxicity of the NPs on HT29 cell line compared to the unmodified NPs. The PC hampers the targeting potential of the studied NPs in HT-29 cancer cells.
	Unmodified NPs (SN-38 conjugated to HA)		
Kim 2008 [44]	Chitosan uncoated PLGA/PVA NPs	Delivery system for paclitaxel in H157 human lung cancer cells	The modification of the nanoparticle surface into positive charge may improve their potential as nanoparticulate drug-delivery carriers, as the chitosan coating slowed the in vitro drug release rate.
	Chitosan (0.2 mg/mL) coated PLGA/PVA NPs		
	Chitosan (0.5 mg/mL) coated PLGA/PVA NPs		
	Chitosan (1.0 mg/mL) coated PLGA/PVA NPs		
Tahara 2009 [34]	Non-PLGA 1000	Uptake of CS PLGA NSs in monolayers of A549 human lung adenocarcinoma cells	Chitosan-modified PLGA NSs are preferentially taken up by human lung adenocarcinoma cells (A549). Chitosan is suitable as a material for surface modification of PLGA nanosystems for intracellular targeting because Chitosan-PLGA NSs increased the interaction between the cell membrane and nanosystems without showing cytotoxicity.
	Non-PLGA 400		
	Non-PLGA 200		
	Chitosan-PLGA 1000		
	Chitosan-PLGA 400		
	Chitosan-PLGA 200		
Yue 2011 [18]	Negatively charged NPs	Evaluation of the effect of surface charge on cellular uptake profiles (rate and amount) and intracellular trafficking	A representative investigation addressing the effects of surface charge on the cellular uptake and intracellular trafficking of chitosan-based NPs on eight cell lines provided directions for optimizing their application in biomedicine
	Neutrally charged NPs		
	Positively charged NPs		
Cheng 2019 [45]	CUR-BCSCs (curcumin (CUR)-loaded biotin-chitosan oligosaccharide-dithiodipropionic acid-curcumin (BCSC) NPs)	Design of chitosan oligosaccharide NPs coated with phycocyanin to enhance the biocompatibility of CUR	The nanomedicine carrier biomaterial of CUR-BCSC@PCs based on chitosan oligosaccharides with multiple functions has provided a new strategy for tumor treatment and exhibited application prospects in cancer therapy as effective drug delivery carriers
	CUR-BCSC@PCs (phycocyanin (PC)-functionalized and curcumin (CUR)-loaded biotin-chitosan oligosaccharide-dithiodipropionic acid-curcumin (BCSC) NPs)		
Buyuk 2020 [46]	NP3 (3.0 mg/mL β-CD, 0.5 mg/mL AMD)	Design of nanoparticulate drug delivery system for the controlled release of amiodarone along with β-cyclodextrin	AMD:β-CD (1:7) mass ratio was the optimal combination. The CD in the solution provided a tighter binding to the NP, resulting in slowing of release. Amiodarone encapsulated in NPs was completely released at the end of 14 days. Amiodarone-loaded chitosan NPs could serve as a model for controlled delivery of many antiarrhythmic drugs
	NP4 (3.5 mg/mL β-CD, 0.5 mg/mL AMD)		
Robles-Planells 2020 [35]	N/P4 (-NH_2_ group of chitosan versus -PO_4_^2−^ group of pDNA), N/P20, N/P28 and N/P40	To confirm the fusogenic activity of ISAV in mammalian cells with chitosan NPs as efficient, low toxicity transfection method.	Chitosan NPs allow the expression of a fusogenically active ISAV fusion protein, which in turn induces cell fusion and cytotoxicity in B16 melanoma cells in vitro. However, its use to treat melanoma tumors induced slight in vivo antitumoral effect in comparison to chitosan treatment.
Rezaei 2020 [47]	Chitosan–lipoic acid nanoparticles (CSLA-NPs)	Design and synthesis of an effective treatment of breast cancer by targeting CD44-overexpressing cells and MT release for systemic delivery.	In vitro experiments revealed that 17α-Methyltestosterone/Hyaluronic acid–chitosan–lipoic acid NPs (MT/HACSLA-NPs) illustrated a sustained drug release in the absence of glutathione (GSH), while the presence of GSH led to fast MT release. HACSLA-NPs also showed high cellular internalization via CD44 receptors, quick drug release inside the cells, and amended cytotoxicity against positive CD44 BT-20 breast cancer cell line as opposed to negative CD44, Michigan Cancer Foundation-7 (MCF-7) cell line.
	Hyaluronic acid chitosan–lipoic acid nanoparticles (HACSLA-NPs)		
Ciro 2020 [48]	DCH-PAM-2Na	Production of novel chitosan NPs and in vitro assessment of release of MTX in simulated physiological conditions (pH 7.4) using these NPs.	The NP systems exhibited encapsulation efficiency ranging from 32% to 66%. These NPs released MTX by an anomalous mechanism. Most NPs exhibited a predominant diffusional release mechanism, whereas PAM-2Na NPs mostly presented a relaxational mechanism. These NPs can be used as a carrier for intravenous methrotrexate release.
	DCH-PAM-2K		
	DCH-PAM-18Na		
	DCH-PAM-18K		
	MTX-DCH-PAM-2Na		
	MTX-DCH-PAM-2K		
	MTX-DCH-PAM-18Na		
	MTX-DCH-PAM-18K		
